# Mitochondria Dysfunction at the Heart of Viral Myocarditis: Mechanistic Insights and Therapeutic Implications

**DOI:** 10.3390/v15020351

**Published:** 2023-01-26

**Authors:** Yasir Mohamud, Boaz Li, Amirhossein Bahreyni, Honglin Luo

**Affiliations:** 1 Centre for Heart Lung Innovation, St. Paul’s Hospital, Vancouver, BC V6Z 1Y6, Canada; 2 Department of Pathology and Laboratory Medicine, University of British Columbia, Vancouver, BC V6T 2B5, Canada

**Keywords:** apoptosis, autophagy, enterovirus, innate immune response, mitochondria, mitophagy, myocarditis

## Abstract

The myocardium/heart is the most mitochondria-rich tissue in the human body with mitochondria comprising approximately 30% of total cardiomyocyte volume. As the resident “powerhouse” of cells, mitochondria help to fuel the high energy demands of a continuously beating myocardium. It is no surprise that mitochondrial dysfunction underscores the pathogenesis of many cardiovascular ailments, including those of viral origin such as virus-induced myocarditis. Enteroviruses have been especially linked to injuries of the myocardium and its sequelae dilated cardiomyopathy for which no effective therapies currently exist. Intriguingly, recent mechanistic insights have demonstrated viral infections to directly damage mitochondria, impair the mitochondrial quality control processes of the cell, such as disrupting mitochondrial antiviral innate immune signaling, and promoting mitochondrial-dependent pathological inflammation of the infected myocardium. In this review, we briefly highlight recent insights on the virus-mitochondria crosstalk and discuss the therapeutic implications of targeting mitochondria to preserve heart function and ultimately combat viral myocarditis.

## 1. Introduction

Viral myocarditis is a leading cause of heart failure in the young populations for which no effective treatments currently exist. Despite significant advancements in research, a therapeutic challenge has persisted largely due to an incomplete understanding of the precise inflammatory reactions that drive acute viral injuries of the myocardium towards chronic inflammation, pathological cardiac remodeling, and cardiac dysfunction [1,2]. Indeed, dysfunction of the biomechanical myocardium presents as a continuum whereby localized injuries can compromise myocyte function at distal sites. Mitochondria, the “powerhouse” of the cells, plays an especially pivotal role in the energy-demanding cardiomyocytes of the continuously beating myocardium. Beyond their roles in energy production, mitochondria also play essential roles in important cellular functions, including metabolism, cell death, and innate immunity [3]. Dysfunctional mitochondria can cause impaired energy generation, dysregulated programmed cell death, and imbalanced innate immune response (i.e., antiviral vs. proinflammatory), ultimately driving viral pathogenesis that underscores myocarditis and its sequelae. 

In this review, we briefly discuss the virus-mitochondria crossfire that takes place during viral infection of the myocardium. We elaborate on the function of mitochondria in sensing and responding to viral infections and discuss strategies utilized by viruses to subvert mitochondrial antiviral signaling efforts. Additionally, we discuss how mitochondrial dysfunction may underscore viral myocarditis. Lastly, we touch upon recent therapeutic approaches that target mitochondria as a strategy for the treatment of viral myocarditis. 

## 2. Viral Myocarditis 

### 2.1. Overview of Viral Myocarditis 

Myocarditis is an inflammatory infiltration of the myocardium that may present through either infectious (e.g., viral, bacterial, fungal, parasitic) or non-infectious (e.g., toxins, pharmacologic sensitivities, immunological syndromes) etiology and can be acute or chronic in nature. Myocarditis can lead to cardiac dysfunction, which may progress to dilated cardiomyopathy (DCM) and heart failure. Among the diverse causes of myocarditis, infections by viral pathogens are the most common etiology [4]. Epidemiologically, the true prevalence of viral myocarditis is challenging to determine but global estimates are currently 10 to 105 per 100,000 individuals with incidence rates of approximately 1.5-1.8 million cases each year [5,6]. Advancements in molecular biology and detection methods have identified specific viruses that are commonly linked to cardiac injury. These viruses include enteroviruses, adenoviruses, influenza viruses, parvoviruses, herpesviruses, and recently, coronaviruses [2,7,8]. Among them, the most comprehensively studied viruses are enteroviruses, particularly coxsackievirus B3 (CVB3) [2].

Although certain clinical features combined with laboratory and imaging findings may be useful in diagnosing viral myocarditis, clinical guidelines such as the Dallas criteria (proposed in 1986) rely on clinicians/pathologists to identify “inflammatory infiltrates of the myocardium with necrosis and/or degeneration of adjacent myocytes, not typical of ischemic damage associated with coronary artery disease.” [9]. Despite its continued use, the Dallas criteria has been criticized for its limitations, including wide sampling error, variations in expert interpretation, inconsistency with other markers of viral infection and immune activation, among others [10]. Indeed, definitive viral etiology of myocarditis will require more precise approaches such as polymerase chain reaction, in situ hybridization and/or immunohistochemical identification that can ultimately help to better inform therapeutics and epidemiological records.

### 2.2. Pathogenic Mechanism 

It is well recognized that the pathogenesis of viral myocarditis is caused by both direct viral damage to the myocardium and indirect lesions secondary to the host immune response [1,2]. During the primary insult (viremic stage of viral myocarditis), cardiotropic viruses directly infect and injure cardiomyocytes through various mechanisms, including the use of viral proteases to disrupt the architecture and function of cardiomyocytes, a strategy that helps to facilitate viral propagation and egress. Enteroviruses encode two proteases, 2A^pro^ and 3C^pro^, which process viral polyprotein into individual structural and non-structural proteins. It has been established that these viral proteases contribute significantly to virus-induced direct injury of the myocardium through cleaving host proteins important for maintaining the normal cardiac architecture and function [2]. For example, enteroviral proteases have been reported to directly target cardiac structural protein dystrophin [11,12,13] and dysferlin [14], as well as cardiac-enriched transcription factor serum response factor [15] to impair cardiomyocyte morphology and cellular function. During the inflammatory phase of viral myocarditis, both virus-induced innate and adaptive immune responses were demonstrated to cause further damage to the myocardium [1,2]. Although the immune response plays a critical role in the host antiviral defense mechanism, an exaggerated or persistent immune response conveys harmful results, contributing to disease-related immunopathogenesis [16]. An appropriate balance between pro-inflammatory defense and anti-inflammatory repair is therefore needed for optimal recovery of viral myocarditis. 

Mitochondrial dysfunction that was commonly observed in both the viremic and inflammatory stages of viral infection has been implicated in the development of viral myocarditis leading to DCM and heart failure [17,18,19,20]. This review will discuss current knowledge about the involvement of mitochondrial dysfunction in the pathogenesis of viral myocarditis, with a particular focus on enterovirus-induced myocarditis that has been extensively investigated in preclinical experimental models. 

## 3. Mitochondria

Mitochondria are double-membraned cellular organelles present in almost all eukaryotic cells. This organelle is essential in various biological processes, and inhibition or dysregulation leads to both regional and systemic homeostatic disturbance.

### 3.1. Mitochondrial Function

The main function of mitochondria in a cell is to produce adenosine triphosphate (ATP) through oxidative phosphorylation (OXPHOS). OXPHOS is a chemiosmotic coupling process between the substrate oxidation and ATP synthesis, occurring in the inner mitochondrial membrane (IMM) through an electrochemical transmembrane gradient generated by the electron transport chain (ETC) [21,22,23]. The ETC reactions are carried out via complex I, II, coenzyme Q, complex III, and complex IV in sequential order with nicotinamide adenine dinucleotide (NAD), succinate, flavin adenine dinucleotide (FAD), ubiquinol, and cytochrome c (cyt c) as their respective electron donors. Mitochondria house their own unique genome termed mitochondrial DNA (mtDNA) that encodes 13 proteins of the ETC [24]. In addition to ATP, mitochondria act as biosynthetic organelles to generate metabolites for the synthesis of macromolecules, such as lipids, proteins, carbohydrates, and nucleic acids [3]. Metabolites from the tricarboxylic acid (TCA) cycle, such as citrate, can be used for the production of cellular fatty acids and sterols, whereas α-keto gluterate (AKG) can be processed to an intermediary metabolite called glutamate, which can drive the production of various amino acids and purines [21,22,23].

Aside from energy production, mitochondria are also involved in diverse cellular processes, including reactive oxygen species (ROS) production, calcium (Ca^2+^) handling, cell death, and signaling integration (see [25,26,27] for a comprehensive review of mitochondrial function). The ROS generated through OXPHOS were initially viewed as “by-products”, but later proven to be physiologically functional by serving as a second messenger in receptor-mediated cell signaling [28]. However, extensive production of ROS can be harmful, causing oxidative damage and ultimate cell death [29]. Mitochondria also play a key role in Ca^2+^ homeostasis through regulating or buffering cytosolic Ca^2+^ concentration. Ca^2+^ is an essential regulator of mitochondrial function and disrupted Ca^2+^ homeostasis has been linked to various pathological conditions. For instance, mitochondrial Ca^2+^ overload can lead to excessive production of ROS, triggering the opening of the mitochondrial permeability transition pore and eventual cell death [30]. 

Mitochondria participate in intrinsic apoptotic pathways by promoting the leakage of pro-apoptotic proteins via an event called mitochondrial outer membrane permeabilization [31,32]. Upon apoptotic stimulation, the Bcl-2 family pro-apoptotic proteins BAX and BAK form channels across the outer mitochondrial membrane (OMM), resulting in the release of cyt c from the mitochondrial intermembrane space to the cytoplasm. Subsequently, cyt c binds apoptosis-protease activating factor 1 to form the caspase-9-activating apoptosome complex, initiating caspase cascade activation and apoptosis [33].

Finally, it is increasingly recognized that mitochondria can serve as a hub for many signaling pathways, through which mitochondria modulate many fundamental cellular functions, including inflammation and immune activation, cell growth, and differentiation [34]. For example, mitochondria have been shown to act as a critical platform for mitochondrial antiviral signaling protein (MAVS)-mediated innate immune signaling pathway, contributing significantly to host defense and inflammation response [35,36]. 

### 3.2. Mitochondrial Dynamics and Quality Control 

Mitochondria are dynamic organelles continually undergoing fusion and fission [37]. The former results in fused organelles that can rescue partially damaged mitochondria, favoring the proper function of ETC for OXPHOS and ATP production upon high-energy demand. The latter is characterized by the division or fragmentation of normal or damaged mitochondria, a process needed for the generation of new organelles and/or mitochondrial quality control to remove terminally damaged mitochondria [38]. Mitochondrial fusion is controlled by three guanosine triphosphatases, i.e., mitofusin 1 and mitofusin 2 (regulating outer membrane fusion), and optic atrophy 1 (modulating inner membrane fusion), while fission process is mainly mediated by dynamin-related protein 1 (Drp1) [37]. Mitochondrial dynamics vary based on cell type and a balance between fusion and fission is crucial for the maintenance of normal mitochondrial function and cellular response to stress [38]. Defects in such processes are implicated in various disorders, including heart disease [38,39,40]. 

Given the detrimental consequences of damaged mitochondria, cells have evolved cellular pathways to sensitively detect mitochondrial injury and efficiently recycle their components/mitochondria through cellular degradation pathways. The dynamic nature of mitochondria not only facilitates fusion and fission processes within the broader mitochondria network but also dictates their capacity to interact with other cellular organelles, such as endoplasmic reticulum and lysosomes to regulate key cellular functions [38]. Among the structures that mitochondria directly interact with, the double-membrane vesicles named autophagosome, a key component of the cellular recycling machinery, is thought to play essential roles in the quality control of mitochondria [41,42,43]. 

**Figure 1:**
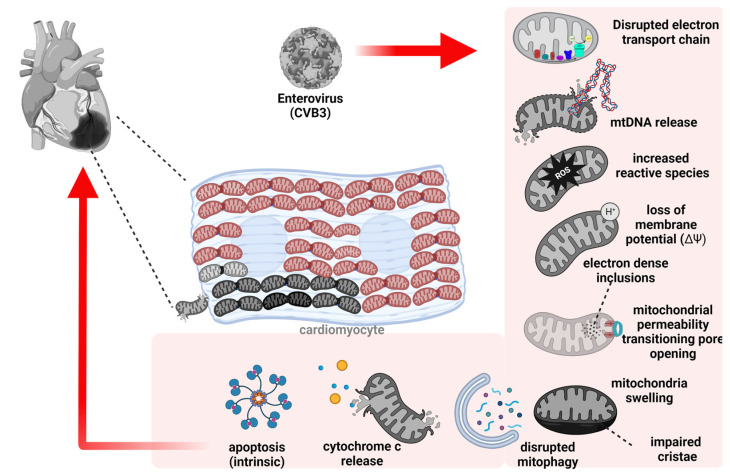
Schematic depiction of virus-induced mitochondrial injury during viral myocarditis. Cardiotropic viruses such as CVB3/EVs, coronaviruses, parvovirus, influenza A virus, Hep C virus, and reovirus can directly injure mitochondria and induce cardiomyocyte pathogenesis. Virus-induced mitochondrial injury includes: disruption of the electron transport chain, release of mtDNA, increased ROS production, loss of membrane potential (ΔΨ), increased electron dense inclusions, mitochondrial permeability transition pore opening, mitochondrial swelling, impaired cristae, disrupted mitophagy, cytochrome c release, and mitochondria-mediated intrinsic apoptosis.

The process of selective autophagic degradation of mitochondria, termed mitophagy, is mediated by autophagic receptors, including sequestosome 1 (SQSTM1), neighbor of BRCA1 gene 1 (NBR1), optineurin, nuclear dot protein 52 (NDP52), and TRAF6 binding protein (T6BP). These receptors bind mitochondrial components (e.g., ubiquitinated mitochondrial proteins following phosphatase and tensin homology-induced kinase 1 (PINK1)/Parkin activation) and target them to autophagosomes via interacting with the autophagosome-anchored protein microtubule-associated protein 1 light chain 3 (MAP1LC3/LC3) [42,43]. Subsequent fusion of autophagosomes with degradative lysosomes results in efficient and specific recycling of mitochondria. In addition to ubiquitin- and PINK/Parkin-dependent mitophagy, several mitophagy receptors that are tethered to the OMM, including FUN14 domain containing 1, BCL2 interacting protein 3 (BNIP3), BNIP3L/NIX, and FK506-binding protein 8, have been shown to interact directly with LC3 to target mitochondria to autophagosomes for degradation [41,44]. Interestingly, recent findings support extracellular release of mitochondria as an alternative mechanism to mitophagy to regulate mitochondria quality control [45]. Figure 1 provides an overview of mitochondrial dysfunction observed following virus infection. 

## 4. Enterovirus-Mitochondria Crossfire

### 4.1. Antiviral Properties of Mitochondria

Among the different organelles of the cell, mitochondria have several distinct features that make them ideal candidates for antiviral defense: (1) injured mitochondria can release antiviral molecules such as ROS to impede viral growth; (2) mitochondria act as gatekeepers of intrinsic programmed cell death/apoptosis that can be leveraged by virally infected cells to limit viral propagation; and (3) mitochondria serve as a central hub for host immune signaling pathways to alarm infected cells and their proximal tissues of ongoing pathogen invasion. 

First, ROS are powerful defense molecules in the arsenal of innate immunity. These highly reactive molecules can exert oxidative damage by altering the host and/or pathogen genomes, proteins, and lipids [29]. In addition, ROS also play a role in non-oxidative innate immunity. For example, overproduction of ROS has been shown to elicit the innate immune response through activating the nucleotide-binding oligomerization domain (NOD)-like receptor (NLR) containing pyrin domain 3 (NLRP3) signaling pathway to counteract invading pathogens [46,47]. NLRP3 is part of the multiprotein complex called the inflammasome that consists of the sensor NLRP3, the adaptor protein ASC (apoptosis associated speck like protein containing CARD), and executioner enzyme pro-caspase 1 that must be activated to initiate inflammasome signaling [48]. Mitochondrial ROS play an essential role in inflammasome activation by facilitating the recruitment of NLRP3 and the adaptor ASC to mitochondria-associated membranes [47]. Moreover, mitochondrial ROS have been reported to induce the expression of MAVS, a key adaptor protein in innate immune signaling, consequently boosting the immune response [49,50]. 

Second, cell death/apoptosis is an evolutionarily conserved mechanism to limit pathogen dissemination [51]. As the last defense against an infection, cells initiate programmed cell death to restrict pathogen replication and propagation and alarm nearby cells of an active infection so that the host defenses can be mounted [51]. 

Third, as alluded to earlier, the OMM where MAVS is situated serves as an ideal platform to induce innate immunity to combat pathogen infection [52]. For example, upon invading, pathogens containing pathogen-associated molecular patterns (PAMPs) can be detected by pattern-recognition receptors (PRRs) [53]. One family of such PRRs is the retinoic acid-inducible gene I (RIG-I)-like receptors (RLRs), including RIG-I and melanoma differentiation associated protein 5 (MDA5). After sensing foreign RNA moieties, the N-terminal caspase recruitment and activation domain (CARD) of these RLRs are exposed to interact with the CARD of MAVS, resulting in MAVS polymerization. Oligomerized MAVS then facilitates downstream activation of interferon regulatory factor 3/7 (IRF3/7) and nuclear factor kappa B (NF-κB), that spearhead the transcription of type I interferon (IFN-I) and pro-inflammatory cytokine [36,53,54]. IFN-I induces the expression of interferon-stimulated genes that favor pathogen detection and restrict replication [55]. Additionally, cytosolic release of mtDNA as a consequence of mitochondrial damage has been demonstrated to trigger the innate immune pathway through activation of the Toll-like receptor 9 (TLR9) [56,57] and/or the cyclic GMP-AMP synthase-stimulator of interferon gene (cGAS-STING) pathways [58,59]. 

### 4.2. Enteroviral Evasion of Mitochondria-Associated Antiviral Immunity

The established role of mitochondria in the regulation of antiviral innate immunity has made it a prime target for viral assault [60]. Enteroviruses, similarly to many other viruses, have evolved effective strategies to target mitochondria for immune evasion. Within the viral components, enterovirus-encoded proteases play a pivotal role in such a mechanism.

The mitochondria-localized MAVS protein has been reported as a key target of enteroviral protease 2A^pro^ and 3C^pro^ to disrupt its antiviral function [61,62,63]. Deletion of MAVS or its upstream sensor MDA5 in mice was shown to increase viral replication and decrease survival of CVB3-infected mice, suggesting an antiviral defense role for MAVS against enteroviral infection [64]. Interestingly, MAVS is cleaved at Q148 by 3C^pro^ and G209, G251, and G265 by 2A^pro^, both instances separating the critical N-terminal CARD domain that is required for MAVS polymerization, RIG-I-MDA5 interaction and downstream IFN-I production from the C-terminal transmembrane domain. In addition to MAVS, other key signaling molecules along the RLR-MAVS-IFN-I antiviral pathway, including MDA5 [61], and RIG-I and IRF7 [65,66,67], are also directly targeted by 2A^pro^ and 3C^pro^, respectively. Collectively, enteroviral proteases cleave multiple critical proteins in the MAVS-associated, mitochondria-centered innate immune signaling to evade immune surveillance. 

**Figure 2:**
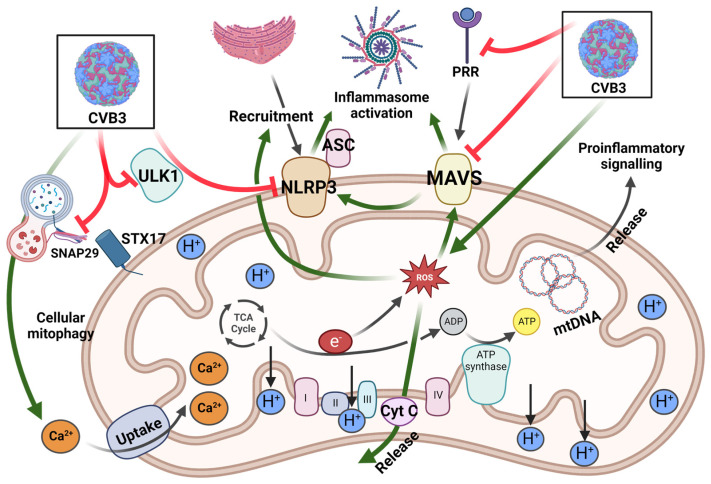
The effects of CVB3 on mitochondrial function. Mitochondrial antiviral signaling protein MAVS and mitochondria recruited ULK1 are key factors during viral recognition and mitophagy initiation, respectively. CVB3 inhibits autophagy initiation and lysosomal fusion by targeting ULK1 and SNAP29/STX17, respectively. ETC-facilitated ATP synthesis involves complex I, II, III, cyt c, and complex VI for coupled reduction-oxidation reactions. MAVS, NLRP3, and ASC all promote inflammasome activation for downstream inflammatory signaling pathways. CVB3 promotes ROS generation, resulting in cyt c release, NLRP3 mitochondrial localization, and induced MAVS expression. Furthermore, viral proteases cause MAVS and ULK1 cleavage, along with enhanced Ca^2+^ influx.

The NLRP3 inflammasome is known as a critical component of the innate immune system against various viral infections, including enteroviral infections, and mitochondria play an important role in NLRP3 activation [68,69]. Mice deficient in NLRP3 have been shown to exhibit delayed viral clearance and more severely impaired cardiac function than wildtype mice, suggesting a protective role at least during early stage of viral infection [68,69]. The antiviral mechanisms of the NLRP3 inflammasome are involved in the promotion of caspase-1-dependent secretion of proinflammatory cytokines (*i.e.,* interleukin 1β (IL-1β) and IL-18), and the induction of inflammatory programmed cell death, termed pyroptosis [70,71]. Upon CVB3 infection, the NLRP3 inflammasomes are activated through diverse mechanisms, including increased production of intracellular ROS and potassium efflux [72]. Beyond ROS, MAVS can also interact with NLRP3 to facilitate its mitochondrial translocation and activation [73]. Moreover, RNA virus-induced, receptor-interacting protein 1 and 3 (RIP1/RIP3)-mediated activation of Drp1, a regulator for mitochondrial fission, has been shown to be necessary for NLRP3 activation by inducing mitochondrial damage [74]. To antagonize the antiviral immunity, enteroviruses have evolved to inactivate the NLRP3 inflammasome by cleaving NLRP3 [68,69] and RIP1/RIP3 [68,75] through the action of enteroviral proteases. Interestingly, cleavage of other members of the NLR family, including NLRP1 and CARD8, by viral proteases was shown to activate antiviral inflammasome signaling [76,77,78]. It is postulated that some NLRs may have evolved to mimic certain regions of viral polyprotein sequences in order to act as an antiviral tripwire that activates upon viral protease-mediated cleavage [78]. A brief outline of CVB3 targeting mitochondrial factors is summarized in Figure 2. 

### 4.3. Mitochondrial Dysfunction Contributing to Pathogenesis of Viral Myocarditis

Progression of acute viral myocarditis towards a chronic inflammatory state and the ensuing sequelae such as DCM or heart failure have been recognized as pathological manifestations of direct viral damage to the myocardium and aberrant immune responses. In particular, mitochondria are emerging as key regulators in cell death, metabolism, and inflammation/immunity, and mitochondrial dysfunction are a driving force for viral pathogenesis in the heart.

#### 4.3.1. Activated Proinflammatory Pathway

The innate immune system acts as the first line of defense against invading viruses. However, it is now clear that excessive and persistent activation of this system can be harmful, leading to chronic inflammation and injuries to the heart. The innate immune system is triggered by enteroviruses via at least 3 classes of PRRs, i.e., TLR, RLR, and NLR, and mitochondria play a central role in such activation [79]. These receptors can recognize viral RNA moieties and elicit a signaling cascade resulting in the production of IFN-I and proinflammatory cytokines. Sustained expression/production of proinflammatory cytokines (e.g., tumor necrosis factor-α, IL-6, IL-1β, and IL-18, among others) has been recognized as a clinical hallmark of viral myocarditis and contributes significantly to disease progression [80,81,82,83]. In line with the vital role of IFN-I in host defense, application of IFN-β during the acute phase of viral myocarditis has been shown to reduce viral load and improve symptoms of myocarditis [84]. In contrast, inhibition of IFN-β at the later stage of disease ameliorates disease progression, suggesting a time-dependent dual function of IFN-β [85]. Likewise, although NLRP3 inflammasome has been shown to play a defensive role during the early phase of a CVB3 infection by removing infected cells and clearing viruses out of the body [68], chronic and sustained activation can be harmful, resulting in immunopathological injury of the myocardium. In CVB3-induced myocarditis mouse model, application of caspase-1 inhibitor or IL-1β neutralizing antibody was demonstrated to ameliorate virus-induced myocardial damage and improve survival [72].

The release of mtDNA is increasingly being recognized as a pathological factor that initiates innate immunity and inflammation in various disease contexts, including cardiovascular pathology [86]. mtDNA is a ~16kb circular DNA that shares inflammatogenic similarities with bacterial DNA (e.g., unmethylated CpGs). Research has revealed that mtDNA can directly activate TLR9 to induce proinflammatory NF-kB signaling, resulting in myocarditis and DCM [56]. Damaged mitochondria are typically recycled by the cellular mitophagy process, but under conditions of autophagy impairment, injured mitochondria may escape recycling and release mtDNA. Interestingly, deletion of the lysosomal DNAse II in cardiomyocytes coupled with an additional stressor, such as pressure-overload, recapitulated mtDNA-dependent myocarditis and DCM [56]. In addition to TLR9, emerging evidence also suggests a crucial role for the cGAS-STING pathway as a sensor of cytoplasmic mtDNA in inducing inflammatory gene expression [87,88].

Studies have established that cGAS-STING activation driven by mislocalized mtDNA contributes to inflammation and tissue damage in myocardial infarction and hypertrophy [89,90,91]. Another example linking cGAS-STING to heart inflammation is the finding that mice lacking three-prime repair exonuclease 1 (Trex1) develop inflammatory myocarditis and DCM [92]. Trex1 is a DNA exonuclease that degrades cytosolic DNA and deletion of Trex1 was shown to activate STING through failure to eliminate self-DNA that has leaked into the cytosol [93,94]. Together, these studies suggest a pivotal role for the cGAS-STING axis in driving cardiac inflammation/dysfunction.

#### 4.3.2. Enhanced Programmed Cell Death

Mitochondrial injury is an important causal factor for programmed cell death such as apoptosis and pyroptosis [31,32]. As a result of disrupted OMM integrity following mitochondrial damage, pro-apoptotic proteins, such as cyt c, in the intermembrane space are released into the cytoplasm to initiate the intrinsic apoptotic pathway. Cyt c is a small 12-kDa heme protein that is loosely associated with the IMM and a crucial element of the ETC and mitochondrial production of ATP [95]. Additionally, cyt c release into the cytosolic compartment is a key cellular signal for programmed cell death/apoptosis. 

The role of mitochondria in regulating physiological programmed cell death/apoptosis can be hijacked by cardiotropic viruses to induce cardiac cell death and enhance viral pathogenesis. Indeed, activating apoptosis is a direct pathway employed by CVB3 to induce cardiomyocyte cell death [96], and such processes have been observed in the myocardium of CVB3-infected mice [97,98] and in cardiac biopsies of viral myocarditis [19,99]. CVB3 infection promotes the production of ROS [100], which facilitate the release of cyt c from mitochondria. In particular, viral protease 2A^pro^ and 3C^pro^ have been shown to activate intrinsic mitochondria-mediated apoptosis [101]. Interestingly, it was demonstrated that 2A^pro^ and 3C^pro^ can also activate the caspase 8-mediated extrinsic apoptotic pathway, suggesting that viral proteases can harness multiple apoptosis pathways to maximize cell death [101]. 

Moreover, CVB3 has been shown to induce other forms of programmed cell death, such as the inflammatory pyroptosis [102]. During pyroptosis, activated caspase 1 cleaves gasdermin D allowing it to form non-specific pores within the plasma membrane of infected cells that ultimately facilitate the release of proinflammatory IL-1β and IL-18 cytokines. In addition, CVB3 and other enteroviruses have been shown to directly activate pyroptosis through viral protease-mediated cleavage of NLRs such as NLRP1, NLRP3, and CARD8 [72,76,77].

#### 4.3.3. Impaired Mitochondrial Quality Control

The high energy demands required to fuel continuous myocardium contractions place cardiomyocytes at an increased risk of oxidative stress and mitochondrial dysfunction. Consequently, quality control processes such as mitophagy, the selective degradation of damaged mitochondria through cellular autophagy, have been shown to play an essential role in cardiomyocyte health. Indeed, impaired autophagy demonstrates pathological misalignments and aggregations of mitochondria and contractile dysfunction of the myocardium [103]. Mechanistic insights in the past decade have revealed unique strategies employed by enteroviruses to subvert protein quality control system in favor of enhanced viral pathogenesis. In particular, CVB3 has been shown to usurp and target host autophagy to facilitate viral growth and impair host quality control [104,105,106]. 

In the myocardium, mitophagy plays a major role in the maintenance of mitochondrial homeostasis and dysfunctional mitophagy has been proven to cause various cardiovascular pathologies [107]. In the context of enteroviral infection, viral proteases are being recognized as critical pathogenic factors that exacerbate disease pathogenesis by targeting diverse signaling proteins involved in mitochondrial quality control [104,105,106].

Unc-51, like autophagy activating kinase 1/2 (ULK1/2), are upstream kinases that function to activate autophagy following diverse stimuli. With respect to mitochondrial quality control, ULK1 plays a key role in linking energy sensors such as AMP-activated protein kinase (AMPK) with mitophagy. Under cellular starvation, AMPK acts upstream of autophagy by directly phosphorylating and activating ULK1 to initiate mitophagy and cell survival [108]. Recent studies have identified ULK1 as a bona fide substrate of viral protease 3C^pro^ [109,110]. It was reported that ULK1 is cleaved after a central glutamine (Q524) residue that bifurcates the N-terminal kinase domain from the C-terminal substrate interaction domain, resulting in impaired autophagy including mitophagy [110]. 

As discussed previously, the selective process of mitophagy is mediated by autophagic receptors, including SQSTM1, NBR1, optineurin, NDP52, and T6BP [41,42,43]. These receptors bridge ubiquitin-modified mitochondrial components with LC3 on the surface of autophagosomes to deliver damaged mitochondria to lysosomes for degradation. Enteroviruses have been demonstrated to cleave SQSTM1, NBR1, NDP52, and T6BP via the proteolytic activities of enteroviral proteases, causing compromised mitochondrial recycling [111,112,113,114,115]. For instance, NDP52 is cleaved by 3C^pro^, resulting in impaired localization to depolarized mitochondria [112,113], while T6BP is processed by 2A^pro^ at a C-terminal glycine (G621) that dispenses the ubiquitin-association domain required for autophagy cargo recognition [113]. Moreover, cleavage of SQSTM1 by 2A^pro^ and NBR1 by 3C^pro^ has been shown to not only cause loss-of-function but also generate dominant-negative cleavage fragments to counteract the function of native proteins in mitophagy [114].

**Figure 3:**
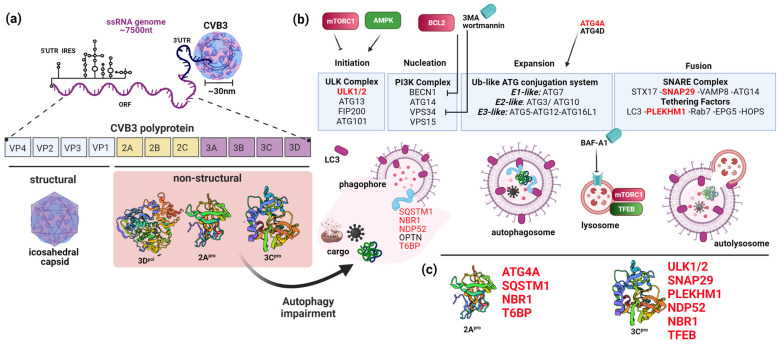
Coxsackievirus B3 (CVB3) Disruption of Cellular Autophagy. (**a**) Schematic depiction of CVB3 structure and proteome architecture. (**b**) Components and process of cellular autophagy beginning with the initiation and nucleation complexes that facilitate autophagosome biogenesis. Maturing autophagosome undergoes expansion via the activity of ubiquitin-like ATG conjugation system. Cargo recruitment inside the developing autophagosomes is mediated by selective autophagy receptors that recognize ubiquitin-modified substrates and bridge with lipidated LC3-II on the autophagic surface. Enclosed autophagosome fuses with lysosomes to facilitate cargo degradation and recycling. (**c**) Viral protease 2A and 3C target key substrates of the autophagy process to impair host cellular recycling and innate immune functions.

Finally, synaptosome-associated protein 29 (SNAP29) is an autophagic soluble N-ethylmaleimide-sensitive factor activating protein receptor (SNARE) protein that functions to bridge autophagosome and/or mitochondria-localized syntaxin-17 (STX17) SNARE protein with lysosome-anchored SNARE protein vesicle-associated membrane protein 7/8 [116]. Following PINK1/parkin activation, mitochondria-derived vesicles (MDVs) fuse with lysosomes in a STX17- and SNAP29-dependent manner [117]. Diverse enteroviruses have been shown to target autophagic SNARES to disrupt the key lysosomal fusion step in the recycling process. For example, enteroviral 3C^pro^ can directly cleave SNAP29, resulting in a bifurcated SNARE that loses the ability to bridge autophagosomes/mitochondria with lysosomes [111,118]. An outline of CVB3-induced perturbations of the host autophagy pathway and recently identified autophagy targets [119,120] are summarized in Figure 3. 

#### 4.3.4. Disrupted Mitochondrial Function 

Indeed, viral infections can significantly alter host cellular metabolism and initiate metabolic reprograming of key energy pathways such as glycolysis, pentose phosphate pathway, and glutaminolysis to ultimately favor viral replication [121]. Moreover, diverse viruses have been shown to alter mitochondrial structure or rewire key mitochondrial energy pathways such as TCA cycle and ETC (reviewed in [121,122]). 

Virus-induced build-up of defective mitochondria may result in either reduced functional capacity and/or enhanced gain-of-toxic function, contributing to the development of viral myocarditis and its progression to DCM. Among the diverse pathophysiological manifestations of viral myocarditis, functional deficits in mitochondrial energy production are the best characterized [17,123,124]. To maintain contractile function and various ATP-dependent ion pumps, the heart requires a high rate of ATP production and turnover. Even minor impairments in mitochondrial ATP synthesis can quickly lead to contractile dysfunction and arrhythmia [125]. Animal studies have implicated that enterovirus-induced mitochondrial injury disrupts essential components of the ETC [123,124]. For example, in a mouse model of CVB3-induced cardiomyopathy, significant changes and reduction of gene expression in mitochondria-associated energy metabolism were observed [124]. Similarly, the activity of cyt c oxidase, the final enzyme in the mitochondrial respiratory chain that helps produce ATP by transferring electrons from cyt c to oxygen, is markedly decreased in isolated cardiac mitochondria in mice infected with CVB3 [123]. Furthermore, in a CVB3 permissive mouse strain, it was found that the activities of all four respiratory chain complexes in mitochondria (I to IV) are significantly reduced and correlated with chronic myocarditis, even in the absence of active viral replication [17]. 

In addition to impaired ATP production, damaged mitochondria can also cause deficits in Ca^2+^ homeostasis. Ca^2+^ plays an important role in muscle contraction and other signaling functions, and its regulation by the mitochondria is critical [30]. CVB3 infection of cardiomyocytes has been shown to cause intracellular Ca^2+^ overload, leading to mitochondrial Ca^2+^ uptake and subsequent cellular apoptosis [126]. This Ca^2+^ overload can also disrupt the tightly regulated electrical activity and contractions of the heart, resulting in potentially lethal cardiac arrythmias [127]. 

Furthermore, impaired mitochondria have also been linked to increased oxidative stress and ROS production. Oxidative stress arising from aberrant ROS production is known to be a major contributor to cardiac injury. Thioredoxin 2 (Trx2) is a key enzyme in mitochondria that helps to maintain cardiac function by scavenging ROS and suppressing ROS production. Clinical studies have shown that the protein level of Trx2 is lower in cardiac tissue from patients with DCM [128]. Mice with a cardiac-specific Trx2 deficiency were found to spontaneously develop DCM and have reduced left ventricular contractile function [128]. In line with the cardioprotective role of antioxidants, overexpression of the mitochondrial antioxidant superoxide dismutase has been shown to have a protective effect on the heart and improve DCM [129]. Indeed, the extreme reactivity of ROS towards various cellular macromolecules including proteins, lipids, and DNA/RNA structures coupled with imbalances in the mitochondrial antioxidant systems, provides a rapid recipe for viral-induced sudden heart failure. 

### 4.4. Mitochondria Fragmentation-Mediated Viral Spread

In the absence of active viral infection, the excess presence of MDVs in circulation has been recognized as a potential marker for the development of cardiovascular disease, particularly in patients with metabolic disorders, such as type 2 diabetes, insulin resistance, and atherosclerosis [130]. Similarly, increased amount of cardiomyocyte-derived extracellular vesicles and mitochondria was detected in circulation following cardiac injury [131]. A key step in MDV production is vesicle fragmentation/division from the mitochondrial network, which has been observed in several clinical settings, including DCM, congenital heart disease, and heart failure [132]. 

Cardiotropic enteroviruses, such as CVB3, are traditionally recognized as non-enveloped viruses. However, they can gain pseudo-envelopes by hijacking cellular lipids, which enhances their infectivity [133]. Furthermore, mitochondrial membranes potentially serve as sources of host-derived envelopes to help the virus egress. It has been demonstrated that CVB3 can replicate and spread in macrovesicles that are released from cardiomyocytes and originate from mitochondrial membranes [134]. Specifically, Sin and colleagues [134] observed that CVB3 capsid protein could be found in association with extracellular microvesicles that contain both mitochondrial markers and LC3, a marker for autophagosomes. The spread of such MDVs may be an underlying feature of cardiac dysfunction, and ability of cardiotropic viruses to exploit these vesicles may exacerbate myocardial pathogenesis. 

## 5. Therapeutic Potential of Mitochondrial Targeting in Viral Myocarditis

There is currently no specific treatment for enteroviral myocarditis, and the management of such a disorder is largely supportive. Given that the pathogenesis of viral myocarditis is caused by both direct viral injury to the myocardium and indirect damage from the host’s immune response, a treatment modality that leverages both antiviral therapeutics and anti-inflammatory agents may be ideal in combatting active infections and suppressing pathological inflammation [1,2]. Although efforts to directly target viral injury through inhibition of virus-encoded proteases have shown promising pre-clinical results, their progress in clinical trials has largely been halted due to drug toxicity concerns [135,136]. Given the importance of host metabolic pathways for efficient viral replication, therapeutic strategies that specifically target metabolic processes usurped by the virus may be worth exploring. It was recently demonstrated that CVB3 enhances glycolytic enzymes to promote viral propagation and that selective inhibition of glycolytic enzymes reduced viral replication [137]. 

As discussed above, dysfunction in mitochondria is associated with the development of various cardiovascular diseases, including viral myocarditis. As a result, targeting mitochondria with drugs that improve their function or increase the number of healthy mitochondria (e.g., inhibiting excessive mitochondrial fission, promoting mitochondrial fusion, or removing damaged mitochondria) in the heart may be a promising approach for treating these conditions. Indeed, early evidence implicates a protective role of antioxidants, such as glutathione peroxidase, in combatting viral propagation and improving disease progression [138,139]. Targeting CVB3-induced ROS with DPI, a chemical inhibitor of NADPH oxidase 4, has also been shown to significantly improve viral myocarditis [140]. Moreover, drugs that regulate mitochondrial dynamics, such as the Drp1 inhibitor, have demonstrated promise in preclinical studies to restrict CVB3-induced cardiac injury [123,134]. Nevertheless, more research is required to fully understand the potential of mitochondria-targeting drugs in the treatment of viral myocarditis. Additional therapeutic candidates that directly target damaged mitochondria, for example through enhanced mitophagy processing or other quality control mechanisms, are also urgently needed. 

In addition to direct viral and mitochondria targeting, therapeutic approaches that address the pathological inflammation of viral myocarditis are also warranted. Despite the recognized role of inflammation in the pathogenesis of myocarditis, early clinical trials using broad immune suppressive drugs failed to show patient benefit [141,142], likely due to suppression of both the protective and deleterious function of the immune response. Recent preclinical evidence implicates that intervention of selective inflammatory pathways, such as the NLRP3 inflammasome pathway, may provide a better therapeutic option than broad immunosuppression [143]. 

Understanding which inflammatory pathways to target at what stage of the disease will be a challenging endeavor, but recent mechanistic insights from other mitochondria-associated diseases such as Parkinson’s, Alzheimer’s, and amyotrophic lateral sclerosis point to a therapeutic potential in targeting mtDNA, particularly through modulation of the DNA sensing cGAS-STING pathway [87,88]. There is growing evidence showing improvements in cardiac inflammation and pathological remodeling by genetically or pharmacologically targeting cGAS-STING [89,90,91]. In addition to dialing down pathological inflammation, an effective therapeutic strategy will also need to enhance regenerative and reparative inflammatory signaling to rejuvenate cardiac tissue back to pre-infection health. Future studies are needed to identify the precise cell types and signaling molecules that provide the best recovery for patients. 

## 6. Concluding Remarks

The lack of efficacious drugs against virus-induced heart injury coupled with recent increases in the emergence of novel viral pathogens with cardiovascular complications (e.g., severe acute respiratory syndrome coronavirus 2 (SARS-CoV-2 and COVID-19-related myocarditis) [7,8,144] are sounding an urgent alarm to develop more effective treatment modalities. The abundance and biological significance of mitochondria within the myocardium and their role in cardiomyocyte function and antiviral innate immunity make them an ideal therapeutic target. Nevertheless, questions remain on how to optimally target mitochondria at various stages of myocarditis progression to improve patient outcomes. For example, (1) can mitochondria be differentially targeted during early and late stages of viral myocarditis to either boost innate antiviral immunity or reduce pathological inflammation, respectively? (2) Which specific mitochondrial targets would facilitate robust antiviral or anti-inflammatory signaling? (3) How can mitochondrial quality control processes be enhanced to bypass viral subversion strategies that limit the function of mitophagy receptors? Indeed, future efforts that aim to better resolve the mitochondria-virus crossfire, through higher resolution surveillance of infected myocardium and mitochondrial dynamics, will be extremely fruitful in helping to address the current challenges and therapeutic void of viral myocarditis. 

## Data Availability

Not applicable.
